# Reliability and Agreement of 3D Trunk and Lower Extremity Movement Analysis by Means of Inertial Sensor Technology for Unipodal and Bipodal Tasks

**DOI:** 10.3390/s19010141

**Published:** 2019-01-03

**Authors:** Rob van der Straaten, Amber K. B. D. Bruijnes, Benedicte Vanwanseele, Ilse Jonkers, Liesbet De Baets, Annick Timmermans

**Affiliations:** 1 REVAL Rehabilitation Research Center, Hasselt University, Agoralaan Building A, 3560 Diepenbeek, Belgium; liesbet.debaets@uhasselt.be (L.D.B.); annick.timmermans@uhasselt.be (A.T.); 2 Department of Orthopaedics, Ziekenhuis Oost-Limburg, Schiepse Bos 6, 3600 Genk, Belgium; amber.bruijnes@zol.be; 3 Department of Movement Sciences, Human Movement Biomechanics, KU Leuven, Tervuursevest 101, 3001 Leuven, Belgium; benedicte.vanwanseele@kuleuven.be (B.V.); ilse.jonkers@kuleuven.be (I.J.)

**Keywords:** inertial sensors, motion analysis, biomechanics, repeatability, functional movement

## Abstract

This study evaluates the reliability and agreement of the 3D range of motion (ROM) of trunk and lower limb joints, measured by inertial measurement units (MVN BIOMECH Awinda, Xsens Technologies), during a single leg squat (SLS) and sit to stand (STS) task. Furthermore, distinction was made between movement phases, to discuss the reliability and agreement for different phases of both movement tasks. Twenty healthy participants were measured on two testing days. On day one, measurements were conducted by two operators to determine the within-session and between-operator reliability and agreement. On day two, measurements were conducted by the same operator, to determine the between-session reliability and agreement. The SLS task had lower within-session reliability and agreement compared with between-session and between-operator reliability and agreement. The reliability and agreement of the hip, knee, and ankle ROM in the sagittal plane were good for both phases of the SLS task. For both phases of STS task, within-session reliability and agreement were good, and between-session and between-operator reliability and agreement were lower in all planes. As both tasks are physically demanding, differences may be explained by inconsistent movement strategies. These results show that inertial sensor systems show promise for use in further research to investigate (mal)adaptive movement strategies.

## 1. Introduction

The use of inertial sensor technology for human motion analysis is increasing. Inertial sensor technology fuses signals from the accelerometer, gyroscope, and magnetometer to estimate the orientation and position of a body segment [1,2]. By applying multiple inertial measurement units on different body segments, three dimensional (3D) joint kinematics can be obtained outside the laboratory [3]. Before an inertial sensor system can be used in clinical practice, it is important to establish the validity and reliability.

Multiple studies have evaluated the validity or reliability of (3D) kinematics measured by an inertial sensor system [4]. However, the majority of these studies only assessed knee joint angles and mainly during level walking. In addition, few studies evaluated the reliability and agreement of the hip, knee, and ankle kinematics measured by an inertial sensor system [5,6,7]. One study also included, besides walking, a squat and a vertical jump [5]. For walking and squatting, acceptable between-day and between-operator reliability were reported, while the reliability and agreement for vertical jump were substantially lower [5]. However, these tasks are less feasible to perform in other populations, such as persons with degenerative joint disease. Moreover, the studies that assessed the reliability only included the lower limb joint kinematics, without including trunk kinematics. Yet, lateral trunk lean has been reported as a (mal)adaptive movement strategy for persons that suffer from functional limitations (e.g., due to pain) [8].

In a recent study, the authors assessed the reliability and agreement of the trunk and lower limb joint kinematics during both the open and closed-chain movement phases of transitional movement tasks, which included walking and physically demanding tasks such as a forward lunge, a sideward lunge, and going up and down stairs [9]. The reliability and agreement of walking were better (i.e., intraclass correlation coefficient (ICC) between 0.4 and 1.0 for all movement phases and standard errors of measurement (SEM) <7.0 degrees) than the reliability of stair negotiation, as stair negotiation had a larger error in all movement plans (i.e., up to 13.5 degrees). The reliability and agreement of flexion/extension and ab/adduction angles during the swing phase and the stance phase of both the forward lunge and the sideward lunge were comparable to that of walking [9]. Currently, little is known about the reliability and agreement of trunk and lower limb joints’ kinematics measured by inertial sensors during unipodal and bipodal lower limb tasks, such as a single leg squat (SLS) and a sit to stand (STS) task.

The SLS is a movement task that is commonly used to identify risk factors for lower limb musculoskeletal injury, as this task provides insight into the lower limb joints’ alignment and altered movement patterns of the trunk, pelvis, and lower limb joints [10]. The STS task is an important activity of daily living and is considered to be essential for independent living in the elderly [11]. Moreover, the STS task is addressed as a functional and valid measure to assess knee function. In this context, the maximum knee extension angular velocity during the STS task is already reported as a parameter to describe knee function. In addition, lateral trunk lean and asymmetrical loading to the contralateral leg during the STS task are recognized as adaptive movement strategies to avoid pain and compensate for quadriceps weakness [11,12]. For both tasks, adequate stability, strength, and neuromuscular control at the level of the trunk, pelvis, hip, and ankle are required for optimal task performance. Given the multidimensionality of the locomotor system, movements can be performed through multiple movement strategies. This implies that the same movement task can be performed in multiple manners by different individuals (i.e., with more or less movement in the different joints and movement planes).

Given that reliability and agreement results are task-specific, this study focusses on the investigation of the reliability and agreement of 3D joint kinematics, assessed by means of an inertial sensor system during a unipodal and a bipodal task. The aim of this study is to investigate the within-session, between-session, and between-operator reliability and agreement of the assessment of the trunk and lower limb joints’ range of motion (ROM) in all movement planes during the performance of the SLS and STS task. Furthermore, the reliability and agreement of the trunk and lower limb joints’ range of motion will be discussed during distinct phases of the movement task (e.g., stand-to-sit and sit-to-stand) in order to make recommendations with regard to task and phase selection for future studies.

## 2. Materials and Methods

### 2.1. Participants

Twenty healthy volunteers, who were selected from a local network of seniors and relatives, participated in the study. As this study is part of a larger study, criteria for inclusion and exclusion are described elsewhere [9]. The study was approved by the ethical committee of the academic hospital Leuven (reference no. s-59857). All participants signed the informed consent before participating in the study. 

### 2.2. Data Collection

#### 2.2.1. Instrumentation

To assess the trunk and lower limb joint kinematics, 15 inertial sensors were used (MVN BIOMECH Awinda, Xsens Technologies, Enschede, The Netherlands). The inertial sensors (MTw Awinda) were placed according to the guidelines provided in the MVN user manual [13]. Therefore, sensors were positioned on the dorsal side of the foot, the medial surface of the tibia (underneath the tibial tuberosity), halfway laterally on the thigh, L5/S1, the dorsal side of the forearm (most distal, between ulna and radius), halfway on the upper arm slight dorsal of the middle line, along the superior border of the scapulae, halfway on the flat part of the sternum, and on the forehead. The inertial sensors were positioned directly on the skin using double-sided adhesive tape, and were secured with a strap in order to preload the inertial sensor and to minimize soft tissue artefacts [14]. The (full-body) joint kinematics were derived from the corresponding MVN BIOMECH software (MVN Studio 4.4, firmware version 4.3.1). In the MVN software, providing the participants’ body dimensions as an input for the full body configuration/model is required, in order to scale the body segments [3]. Before each session, a static N-pose calibration was performed in order to align the sensor’s orientation to the segment’s orientation. The static calibration was done while the participant was standing in an N-pose position; standing in an upright neutral position, with the arms alongside the body, their feet parallel beside each other pointing forward, and making sure that both legs and arms were in a straight line downwards.

#### 2.2.2. Movement Protocol

The movement protocol included the following tasks: SLS and STS movement. A detailed description of the instructions to the participant is provided in Table 1. For the SLS, the participants performed a squat on the right leg (until their maximum knee flexion, while maintaining balance) with their hands on their pelvis. For the STS task, the participants stood in front of the stool, sat down (without looking over their shoulder), and stood back up again. The stool height was set at the participants knee height. Additionally, the task was demonstrated by the operator and each participant received practice trials to familiarize themselves with the task. All participants performed the protocol three times (two tasks, five repetitions per task) on two different days.

On the first day, one session was performed by “Operator 1” and the second by “Operator 2”, to determine the within-session and between-operator reliability and agreement. Between the two sessions, the inertial sensors were removed and a half hour break was implemented in order to rest and allow skin and strap marks to disappear. On the second test day, which was, on average, nine (SD = 4) days later, the participants returned to the lab and the protocol was repeated by the same operator, in order to determine the between-session reliability and agreement. More details on the assessment are described elsewhere [9]. Both operators, experienced in conducting motion analysis studies, gave structured and concise instructions to the participants (Table 1) and evaluated task execution. If task execution deviated from the instructions, an additional trial was recorded.

### 2.3. Data Analysis

**Figure 1:**
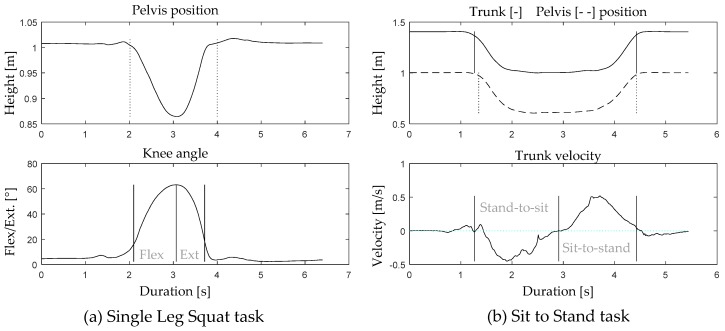
(**a**). Definition of the flexion and extension movement phase during the single leg squat (SLS) using the pelvis position and knee flexion/extension angle; (**b**) definition of the stand-to-sit and sit-to-stand (STS) phase, using the trunk and pelvis position and the trunk velocity.

Three-dimensional joint kinematics of the trunk, pelvis, hip, knee, and ankle were directly extracted from the MVN software. Within the MVN software, the body segments of the anatomical model are defined based on the recommendations of the international society of biomechanics [15]. In this model, the *x*-axis represents frontal plane joint movements (abduction/adduction), the *y*-axis represents transversal plane joint movements (internal/external rotation), and the *z*-axis represents sagittal plane joint movements (flexion/extension). Movement cycles were divided into sub-phases, using a custom written algorithm in Matlab (2016b, Mathworks, Inc., Natick, MA, USA). The SLS was divided in two sub-phases: knee flexion and extension. The flexion phase was defined as the period when the pelvis position was moving down and in which the knee flexion/extension angle exceeded the threshold of 25% of maximum knee flexion until maximum knee flexion. The extension phase was defined as the period in which the pelvis position was moving back up and the knee extension reached the 25% knee flexion/extension angle threshold (Figure 1a).

For the STS task, a stand-to-sit and a sit-to-stand phase were defined. The stand-to-sit phase started when the trunk or pelvis position moved downwards (first one that was going down was used) and stopped when the person was seated and the trunk started to move forward again (change in velocity from negative to positive). The sit-to-stand phase started from the instant the trunk moved forward (velocity is positive) and ended when the trunk and pelvis position returned to the initial start position (Figure 1b).

The trunk, hip, knee, and ankle angles were normalized to 0% to 100% for each phase. For each phase, the minimum and maximum angle was determined per joint, in order to calculate the range of motion (ROM). The ROM was defined as the absolute difference between the minimal and maximal joint angle. The ROM was selected because the start and end point of each movement phase contained full extension (especially for the sagittal plane angles). From a clinical perspective, the evolution in joint ROM is furthermore of interest in order to evaluate treatment efficacy or to compare between groups. The first repetition was deleted from the analysis, as it could be disturbed by initiation strategies. Furthermore, all trials were visually inspected for incorrect data resulting from technical errors and only data from the right limb were used for data-analysis.

### 2.4. Statistical Analysis and Data Interpretation

#### Statistical Analysis

Statistical analysis was performed using SPSS (version 25, IBM Corporation, Amonk, NY, USA). The reliability of the ROM in all movement planes and joints was determined based on the intraclass correlation coefficient (ICC), including the 95% confidence interval. Agreement was evaluate using the standard error of measurement (SEM), based on the square root of the mean square error term of the analysis of variance (ANOVA) and the minimum detectable change (MDC) between two sessions, using the SEM (MDC = SEM × 1.96 × √2) [16]. A proportional SEM (%SEM) was calculated to provide information on the magnitude of the SEM relative to the measured ROM, by expressing the SEM relative to the mean (%SEM = (SEM/mean) × 100%). Single repetition data were used to calculate the within-session (intra-operator) reliability (ICC2,1) and agreement. Averaged data from four repetitions per day were used to calculate the between-session (intra-operator) reliability (ICC2,k) and agreement. Averaged data from four repetitions per operator were used to calculate the between-operator (inter-operator) reliability (ICC2,k) and agreement. ICCs ≥ 0.90 were considered as excellent, 0.70–0.89 as good, 0.69–0.40 as acceptable, and <0.40 as low [17].

Because the reliability and agreement results are affected by the magnitude of the ROM, a distinction was made between sagittal plane movement (i.e., flexion/extension) and frontal/transverse plane movement (i.e., ab/adduction and in/external rotation) when outlining the results. During both the SLS and the STS task, flexion/extension movements had the largest ROM in the different lower limb joints and/or at the trunk. However, to achieve a full flexion/extension movement in the different lower limb joints, conjunct movements that are mechanically coupled to flexion/extension kinematics (i.e., movements in the transversal and frontal plane) are required. Therefore, the flexion/extension ROM of the trunk and lower limb joints is referred to as “main-movement” and the ab/adduction and the in/external rotation ROMs are referred to as “conjunct-movement”.

## 3. Results

### 3.1. Participants

Twenty healthy participants, 9 males and 11 females with an average age (SD) of 62.7 (8.5) years and body mass index of 24.4 (3.6) kg/m^2^, were enrolled in the study.

### 3.2. Reliability and Agreement

In the following paragraph, the reliability and agreement of the trunk and lower limb joints’ 3D ROM will be reported. The within-session, between-session, and between-operator reliability and agreement of the main movement ROM are presented first, followed by the conjunct movement ROM for both tasks. Individual results (ICC, SEM, and MDC) of the within-session, between-session, and between-operator can be found in the Supplementary Materials (Tables S1 and S2). Additionally, waveforms of each task, phase, and movement plane are presented in the Supplementary Materials (Figures S1 and S2).

#### 3.2.1. Main Movement

**Figure 2:**
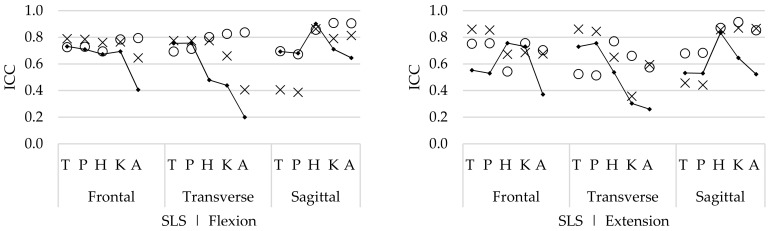
Within-session (▪), between-session (×), and between-operator (ο) intraclass correlations (ICCs) of the trunk (T), pelvis (P), hip (H), knee (K), and ankle (A) range of motion (ROM) of all rotations, in the flexion phase (**left**) and the extension phase (**right**) during the single leg squat (SLS) task.

For the SLS task, the within-session reliability of the hip flexion/extension ROM was good for the flexion phase (ICC 0.90) and the extension phase (ICC 0.83). The ICCs for the trunk, pelvis, knee, and ankle ROM were acceptable to good (ICC 0.52–0.71) in both movement phases (Figure 2). Between-session and between-operator reliability were good to excellent (ICC 0.81–0.91) for the hip, knee, and ankle flexion/extension ROM, in both movement phases, while the ICCs of the trunk and pelvic ROM ranged from low to acceptable (ICC 0.39–0.70) in both movement phases (Figure 2).

**Figure 3:**
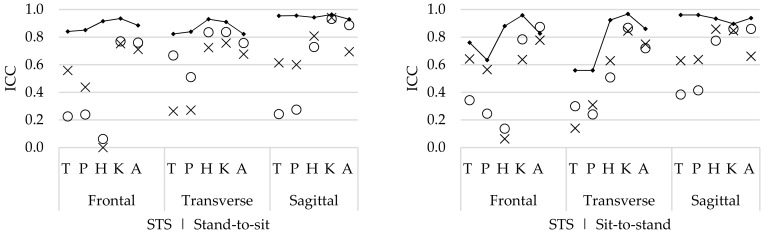
Within-session (▪), between-session (×), and between-operator (ο) intraclass correlations (ICCs) of the trunk (T), pelvis (P), hip (H), knee (K), and ankle (A) range of motion (ROM) of all rotations, in the stand-to-sit (**left**) and the sit-to-stand phase (**right**) during the sit to stand (STS) task.

For the STS task, the within-session ICCs for trunk, pelvis, hip, knee, and ankle flexion/extension ROM were good to excellent (ICC 0.90–0.96). Between-session and between-operator ICCs were good to excellent for the flexion/extension hip and knee angles (0.73–0.94) and acceptable to good for the flexion/extension ankle angles (ICC 0.66–0.89) in both movement phases (Figure 3). For the trunk and pelvic ROM, between-session reliability was acceptable (ICC 0.60–0.64), while the between operator reliability was low (ICC 0.24–0.41) in both movement phases (Figure 3).

#### 3.2.2. Conjunct Movement

For the SLS task, the ab/adduction ROM within-session ICCs of the trunk, pelvis, hip, and knee ROM were acceptable to good (ICC 0.53–0.76), while the ICCs of the ab/adduction ankle ROM were low (ICC 0.41, 0.37) for both movement phases (Figure 2). Between-session and between-operator ICCs were acceptable to good (ICC 0.54–0.86) for all joints in both movement phases.

For the in/external rotation, good within-session reliability (ICC 0.73–0.76) was found for the trunk and pelvic ROM, while the ICCs for the in/external rotation of the hip, knee, and ankle ROM were acceptable to low (ICC 0.20–0.54). Between-session and between-operator ICCs were acceptable to good (ICC 0.41–0.76) for all joints in both movement phases (Figure 2), apart from the ICC of the in/external rotation knee ROM (ICC 0.36).

For the STS task, the ab/adduction ROM within-session ICCs were good to excellent in all joints for the stand-to-sit phase (ICC 0.84–0.94) and for the sit-to-stand phase (ICC 0.76–0.96), except for the pelvic ROM reliability, which was acceptable (ICC 0.44, 0.63). The between-session and between-operator ICCs for the knee and ankle ROM were acceptable to good in both movement phases (ICC 0.64–0.87), while the ICCs of the trunk, pelvis, and hip ROM were low to acceptable (ICC 0.0–0.64) in both movement phases (Figure 3).

The within-session reliability of the in/external rotation ROM of all joints was good to excellent (ICC 0.82–0.93) for the stand-to-sit phase. For the sit-to-stand phase, only the hip, knee, and ankle ROM showed good to excellent within-session reliability (ICC 0.86–0.97), while the reliability of the in/external rotation trunk (ICC 0.56) and pelvic (ICC 0.56) ROM was lower. The between-session reliability of the hip, knee, and ankle in/external rotation ROM was acceptable to good (ICC 0.63–0.85), while the ICCs for the trunk and pelvis were low (ICC 0.14–0.31) in both movement phases. Regarding the between-operator reliability, acceptable to good results (ICC 0.51–0.84) were found for all in/external rotation ROM in the stand-to-sit phase (Figure 3). For the sit-to-stand phase, only good results were found for the knee and ankle ROM (ICC 0.87, 0.72). For the trunk, pelvis, and hip ROM, acceptable to low between-operator ICCs were found (ICC 0.24–0.51).

#### 3.2.3. Agreement

**Figure 4:**
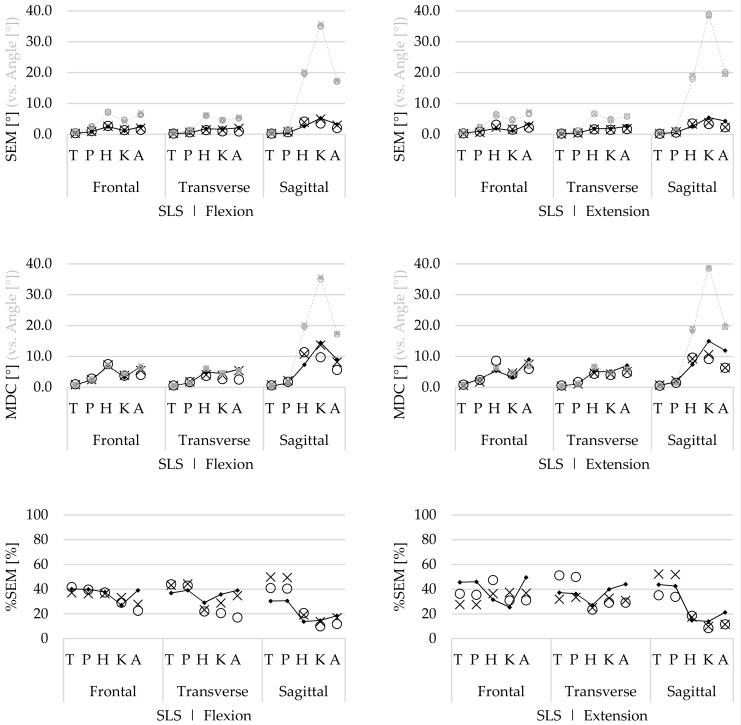
Within-session (▪), between-session (×), and between-operator (ο) standard errors of measurement (SEM), minimum detectable change (MDC), and proportional SEM (%SEM) of the trunk (T), pelvis (P), hip (H), knee (K), and ankle (A) range of motion (ROM) of all rotations, in the flexion phase (**left**) and the extension (**right**) phase, during the single leg squat (SLS) task. The grey in the mean ROM of all joints indicates the (proportional) difference with the SEM and MDC.

For the SLS task, comparable within-session, between-session, and between-operator agreement (Figure 4) was found for both movement phases. The highest SEM and MDCs were reported for the hip, knee, and ankle flexion/extension ROM (SEM 2.6–5.4°, MDC 5.6–14.4°). However, the ROM of these movements was the highest (Figure 4), and thus the proportional SEM of these flexion/extension ROMs was lower (%SEM 8.5%–21.3%). For the trunk and pelvic flexion/extension ROM, as well as for the ab/adduction and in/external rotation ROM, higher agreement was found (SEM 0.1–3.2°, MDC 0.4–7.5°). However, the ROM of these angles was lower and thus the proportional SEM was higher for the ab/adduction (%SEM 22.5%–49.6%) and in/external rotation angles (%SEM 17.1%–51.2%).

**Figure 5:**
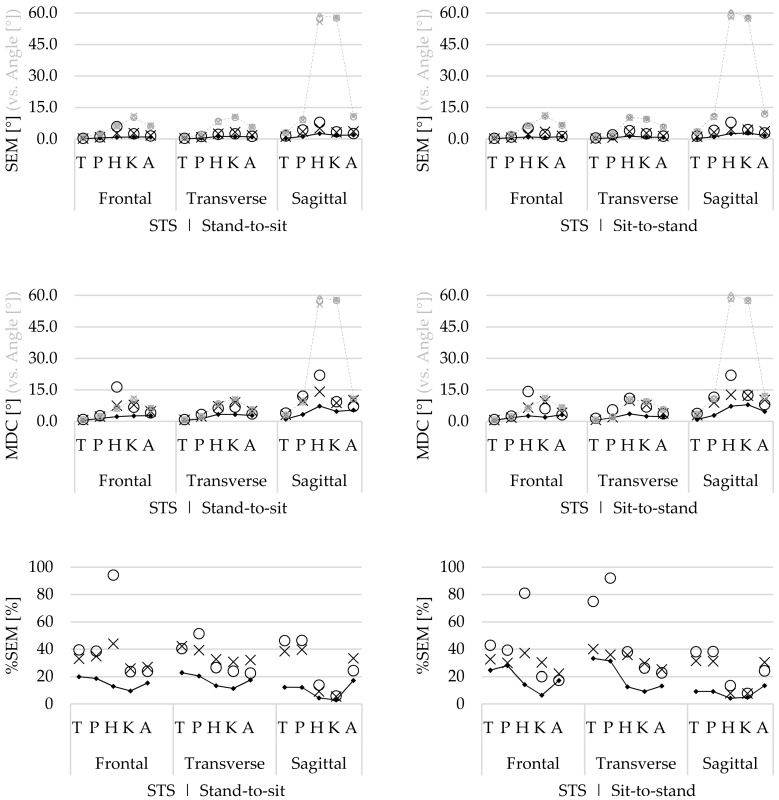
Within-session (▪), between-session (×), and between-operator (ο) standard errors of measurement (SEM), minimum detectable change (MDC), and proportional SEM (%SEM) of the trunk (T), pelvis (P), hip (H), knee (K), and ankle (A) range of motion (ROM) of all rotations, in the stand-to-sit (**left**) and the sit-to-stand phase (**right**), during the sit to stand (STS) task. The grey in the mean ROM of all joints indicates the (proportional) difference with the SEM and MDC.

For the STS task, the within-session agreement was higher compared with the between-session and between-operator agreement in both movement phases (Figure 5). Similar to the SLS, the hip, knee, and ankle flexion/extension ROM had the lowest agreement (SEM 0.4–7.9°, MDC 1.1–21.0°). However, for the hip and knee angles, the proportional SEM was lower (%SEM 3.0%–13.8%) compared with the results for the ankle joint (%SEM 13.5%–33.4%). Highest %SEMs were found for the between-operator ab/adduction hip ROM (%SEM 94.3%, 81.0%) during both movement phases and in/external rotation trunk (%SEM 74.9%) and pelvic (%SEM 92.1%) ROM during the sit-to-stand phase (Figure 5).

## 4. Discussion

This study aimed to assess the within-session, between-session, and between-operator reliability and agreement of the trunk and lower limb joints’ 3D ROM during the SLS and STS task. Additionally, the reliability and agreement of the different movement phases of the SLS and the STS tasks were reported. For the SLS task, only small differences were found between the flexion and extension phase, in all movement planes. In contrast, during the STS task, larger differences in multiple movement planes were obtained between both movement phases.

### 4.1. Interpretation of Study Results

#### 4.1.1. Main Movement

The main movements include the flexion/extension ROM of the trunk and lower limb joints. For the STS task, the within-session ICCs were good to excellent for the hip, knee, and ankle ROM in both movement phases. Between-session and between-operator ICCs were also high for the hip, knee, and ankle ROM in both movement phases, but slightly lower compared with the within-session reliability (Figure 5). The trunk and pelvis ROM within-session ICCs were acceptable, but lower compared with the hip, knee, and ankle ROM. Also, for the between-session and between-operator reliability, the trunk and pelvis ROM was lower.

For the SLS, the within-session reliability of the hip ROM was high; however, the reliability of the knee and ankle ROM was lower in both movement phases. These lower ICCs indicate that there is more natural movement variability in these joints. This is also confirmed by the within-session SEM (Figure 4), which show greater error in the knee flexion/extension ROM during the SLS. These differences in variability might be explained by the fact that the knee flexion angle (depth of the SLS) had no clear endpoint, as the depth of the SLS was standardized based on the maximum knee flexion without losing balance. In contrast, the stool height (at knee height) was a clear endpoint during the STS task, and thus results in less variation between repetitions.

In such cases, thorough instructions and task explanation are critical. Given the fact that the between-session and between-operator reliability were higher, it is expected that these differences were related to natural movement variability in trial-to-trial repetition.

#### 4.1.2. Conjunct Movement

The conjunct movements contain the ab/adduction and in/external rotation ROM in all joints. For the STS task, the within-session reliability was good and higher for all ROMs, compared with the between-session and between-operator ICCs. Differences in the between-session and between-operators ICCs were present for the STS task, but these differences were not consistent (Figure 3). For the SLS task, the within-session reliability was relatively low (compared with the STS task) for all joints. In contrast to the STS task, the between-session and between-operator ICCs of the SLS task were comparable or higher than the within-session ICCs for all joints.

The within-session agreement results confirm that the STS task was performed with less natural movement variability compared with the SLS task, because the SEM and %SEM were systematically lower for the STS (Figure 5) compared with those for the SLS task (Figure 4). However, compared with the within-session agreement, the between-session and between-operator agreement for the SLS remain similar, while the agreement for the STS task decreases. This demonstrates that although there is natural variability during the performance of repetitive SLS movements, this remains constant, as well as between sessions and operators. Furthermore, during the SLS, the ICCs increase for both the between-session and between-operator reliability compared with the within-session ICCs. Therefore, it is assumed that the natural movement variability is inconsistent between trial repetitions. As for this study, healthy participants were recruited, and it seems that they apply different movement strategies to perform the SLS between different repetitions, especially for the hip, knee, and ankle ab/adduction ROM and in/external rotation ROM (Figure 2). However, by doing this inconsistently, the average over multiple trials will cancel out these differences.

In contrast to the SLS, the agreement decreases for the between-session and between-operator reliability for the STS task. Especially for the joints that include the pelvic sensor in the calculation of the joint angle (i.e., trunk, pelvis, and hip angles), the SEM increases and ICCs are generally lower. During data collection, it was noticed that the position of the pelvis sensor (especially during a dynamical task involving a lot of hip flexion such as the STS task) was easily altered by the participants’ shorts, which moved over or against the sensor, and thus potentially affected the orientation of the sensor (which is used for the calculation of multiple joint angles including the trunk and hip). Moreover, because the between-operator %SEM is ≥75% for the hip, trunk, and pelvic ROM during the STS task, it is presumed that there are methodological differences between sessions and operators.

In comparison, no studies investigated the reliability and agreement of trunk, pelvis, and lower limb joint kinematics during an SLS or STS task by means of an inertial sensor system. Only one study reported acceptable to good reliability and agreement of the flexion/extension kinematics from the lumbar spine and hip joint during functional movement control tasks (including a stand-to-sit-to-stand task) in healthy persons using inertial sensors [18]. In addition, a recent study reported the reliability and agreement of the SLS task by means of an electromagnetic tracking system [19]. The reported reliability and agreement of the trunk, pelvis, hip, and knee joints were higher compared with the results in the present study. However, in the study of Nakagawa and colleagues, only young healthy participants (20.8 ± 1.7 years) were included. As it is expected that the lower ICCs for the SLS in the present study are caused by natural movement variability, it appears that this variability is not present in a younger population. Additionally, in comparison with other functional tasks, assessed by means of an inertial sensor system, the SLS task showed good reliability and agreement, especially for the between-session and between-operator reliability, whereas the reliability and agreement of the STS task were comparable to other functional tasks [5,9].

#### 4.1.3. Differences in Movement Phases

The SLS task showed slight differences in reliability or agreement between the flexion and extension phase. For the STS task, however, differences between movement phases were more obvious. For the sit-to-stand phase, the within-session ICCs of the trunk and pelvic ab/adduction and in/external rotation ROM were substantially lower compared with the stand-to-sit phase (similar differences were also observed in the agreement metrics). This indicates that both tasks show lower reliability and agreement in the return phase. Similar findings were reported for other functional movement activities such as the forward and sideward lunge. In these tasks, lower reliability was also reported for the swing backwards compared with the swing forward [9], similar to the swing backward during the forward and sideward lunge, for the sit-to-stand phase and the extension phase of the SLS. This is potentially caused by the fact that extension of the knee joint (pushing the body back up) requires more motor control and force compared with the opposite movement.

#### 4.1.4. Recommendations

For the SLS, it is recommended to use the averaged data over multiple (in this study four) repetitions, as the between session ICCs were substantially better than the within-session ICCs. Furthermore, no differences were observed between phases, thus both phases are reliable for use in the assessment of the SLS task. For the STS task, the individual repetitions showed good reliability and agreement. The averaged repetitions were lower, but not consistently different from each other, which indicates that there are no systematic differences between test sessions or operators. On the contrary, the averaged data showed lower reliability (and agreement) compared with the within-session data, which indicates that there are individual differences in task execution between test occasions. These differences occur predominantly in the angles (trunk, pelvis, and hip joints) that are determined based on position and orientation of the pelvic sensor. Therefore, in the case of a longitudinal data collection, it is recommended that this data collection is performed by one (the same) operator during all sessions. Furthermore, the sensor positioning should be standardized to the highest extent and during the calibration procedure, the participant should be positioned passively in the correct position in order to improve the sensor-to-segment alignment [13,20]. Proper strapping of the inertial sensors is required to reduce soft tissue artefacts and the proximity of ferromagnetic materials should be avoided in order to avoid magnetic disturbances [3,14]. Finally, it is recommended to ensure that the position and orientation of the pelvic sensor is not affected by the clothing during task execution.

### 4.2. Limitations and Future Research

As stated above, the within-session ICCs from the SLS were relatively low, whereas the between-session and between-operator ICCs were substantially higher. One reason for this is that the SLS is a clinical test to identify kinematic risk factors for lower limb musculoskeletal injury. It is important to perform this test according to an individual’s potential, in order to increase the clinical utility of the task. Another potential reason for this result could be that the healthy persons assessed in this study are not aware of the fact that they use multiple movement strategies in order to successfully achieve the SLS (i.e., movement redundancy). The authors assume that in the cases in which persons with pathology (e.g., degenerative disorders) are measured during the performance of an SLS, the within-session results might be higher. As it has been shown that movement variability decreases in the cases of pathology, and these persons adapt to a specific movement strategy [21]. Therefore, to achieve the functional movement task, persons with a painful condition at the knee or hip joint might only rely on one specific movement strategy (i.e., decreased movement redundancy) as a result of their pain, which leads to higher within-session ICCs. Future studies are needed to examine this in a patient population. Furthermore, in this study, only one leg was analysed. However, it is known that movements of the non-weight bearing limb affect the trunk and lower limb joint kinematics [10]. Therefore, future studies should not only investigate the weight bearing limb, but also focus on the non-weight bearing limb.

Within the present study, trunk and lower limb joint kinematics were recorded using a commercially available inertial sensor system (MVN BIOMECH Awinda, Xsens Technologies). The results thus need be interpreted within the constraints imposed by the specificity of the technology and calibration method. For joint angle calculation, inertial sensor technology generally relies on signal fusion of an integrated accelerometer and gyroscope signal and the magnetometer [3]. It is known that (double) integration of the accelerometer and gyroscope signal, in order to determine sensor position and orientation, suffers from integration drift. To compensate for this drift, the magnetometer is used as a reference [2]. Nevertheless, the magnetometer is easily disturbed by ferromagnetic materials or electronic devices, which invalidates the estimated position and orientation of the sensor and may affect the sensor-to-segment alignment during the static calibration [22]. In order to reduce these errors, it is recommended to avoid the proximity of ferromagnetic materials. New methods have been developed that overcome these constraints by tracking motion without using the magnetometer [22,23]. However, these methods still need further development before they can be implemented in clinical research. In addition, careful positioning of the inertial sensors is crucial to maximally reduce soft-tissue artefacts, for example, due to muscle contraction, by positioning the sensor preferably on bony landmarks in order to minimize motion between the sensor and the bones. Proper strapping of the inertial sensor minimizes this movement artefact, but to date, it is impossible to completely avoid these artefacts.

For the present system, the initial static calibration procedure is sensitive to the predefined positioning and alignment of the inertial sensors [24]. The participant should thus be positioned passively in the correct position in order to improve the sensor-to-segment alignment [3,14]. As the measurements in our study were conducted by well-trained operators, we are confident that these effects were maximally reduced, but were likely still present in the data and may affect data interpretation. The results of the present study must be interpreted while keeping this in mind. Future research should focus on improving the algorithms for the sensor-to-segment alignment and calibration procedures, in such a manner that the user is not bounded to a predefined sensor positioning, and so that a non-expert can use such a system. The initial results of studies that facilitate measurements with less accurate sensor alignment are promising [25,26,27]. In the long term, this could lead to new opportunities to use inertial sensor technology outside the laboratory setting, such as in clinical practices or at home situations, which leads to a new range of opportunities.

Finally, as the present study demonstrated the reliability and agreement of functional movement tasks, future research should focus on the accuracy of the inertial sensor system and compare the trunk and lower limb joints’ kinematics measured by means of an inertial sensor system against an optoelectronic system (gold standard). In cases in which the accuracy of the kinematics from the inertial sensor system is known, it can potentially be used to assess (mal)adaptive movement strategies in persons with pathology.

## 5. Conclusions

Ambulatory motion analysis of the trunk and lower limb joints’ ROM (using inertial sensor technology) had good reliability and agreement for both the flexion and extension phases of the SLS, for both main and conjunct movements (when using averaged data over multiple trials). The reliability and agreement for the main movements during the STS task were also good. However, care should be taken regarding the position of the pelvis sensor during the sit-to-stand phase, as this might affect the reliability of conjunct movements. Further research should investigate the reliability and agreement in persons with functional limitations and focus on the comparison of joint kinematics measured by means of an internal sensor system with an optoelectronic system. This validation is necessary before the inertial sensor system can be applied in clinical practice for the identification of (mal)adaptive movement strategies.

## Figures and Tables

**Table 1:** Detailed description of the instructions to the participants.

Task		Instruction
Single Leg Squat (SLS)	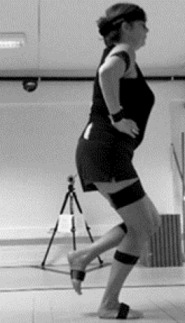	Stand still with feet shoulder width apart and put your hands on the pelvis. Shift the weight to the right and lift the left foot from the ground by performing a flexion of the left knee. When standing on one leg with full knee extension, squat on the right leg as deep as possible but remain balanced and make sure the left leg is not contacting the ground. When maximal flexion is reached, extend the right knee and when the right leg is fully extended, place your left foot again down on the floor.
Sit to stand (STS)	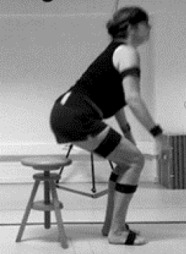	Stand with your back towards the stool with the feet shoulder width apart and with the arms hanging alongside of the body. Sit down without looking over your shoulder, remain seated (like on a chair), and stand up again (without swinging your arms).

## Supplementary Materials

The following are available online at http://www.mdpi.com/1424-8220/19/1/141/s1. The individual results from the ICCs, SEMs, and MDCs from all tasks are provided in Tables S1 and S2. Waveforms for each task, phase, and rotation are provided in Figures S1 and S2.
