# Supplementary material for: Reliability and Agreement of 3D Trunk and Lower Extremity Movement Analysis by Means of Inertial Sensor Technology for Unipodal and Bipodal Tasks
Source: Sensors (Basel). 2019 Jan 3;19(1):141. doi: 10.3390/s19010141 (PMC6339112; doi:10.3390/s19010141)

Table S1: Within-session, between-session and between-operator reliability (ICC and confidence intervals (CI)) and agreement (SEM, MDC, %SEM and %MDC) from the single leg squat (SLS) task during both movement phases (Flexion and Extension phase).

			Flexion							Extension						
			ICC	CI	mean (± SD)	SEM	MDC	%SEM	%MDC	ICC	CI	mean (± SD)	SEM	MDC	%SEM	%MDC
within-session	Frontal	Trunk	0,73	0,56 - 0,87	0,7 ± 0,5	0,3	0,8	40,2	111,3	0,55	0,34 - 0,76	0,7 ± 0,5	0,3	0,9	45,6	126,5
		Pelvis	0,71	0,53 - 0,86	2,1 ± 1,5	0,8	2,4	39,9	110,5	0,53	0,31 - 0,74	2,1 ± 1,4	1,0	2,7	46,1	127,9
		Hip	0,67	0,48 - 0,83	6,6 ± 4,3	2,5	6,9	37,8	104,6	0,76	0,59 - 0,88	6,1 ± 3,8	1,9	5,3	31,5	87,4
		Knee	0,69	0,51 - 0,85	4,3 ± 2,1	1,2	3,2	27,2	75,3	0,73	0,59 - 0,87	4,6 ± 2,2	1,2	3,2	25,4	70,4
		Ankle	0,41	0,18 - 0,65	6,2 ± 3,1	2,4	6,7	39,2	108,7	0,37	0,16 - 0,62	6,5 ± 4,2	3,2	9,0	49,6	137,5
	Transverse	Trunk	0,76	0,59 - 0,88	0,4 ± 0,3	0,2	0,4	36,9	102,3	0,73	0,56 - 0,87	0,4 ± 0,3	0,1	0,4	37,3	103,3
		Pelvis	0,76	0,6 - 0,88	1,3 ± 1	0,5	1,4	39,2	108,6	0,76	0,59 - 0,88	1,1 ± 0,8	0,4	1,2	36,3	100,5
		Hip	0,48	0,25 - 0,71	6,2 ± 2,4	1,8	5,0	29,1	80,7	0,54	0,32 - 0,75	6,7 ± 2,7	1,8	5,0	27,0	74,9
		Knee	0,44	0,22 - 0,68	4,5 ± 2,1	1,6	4,4	35,8	99,3	0,30	0,09 - 0,57	4,5 ± 2,2	1,8	5,0	40,0	110,8
		Ankle	0,20	0 - 0,47	5,4 ± 2,3	2,1	5,9	39,1	108,3	0,26	0,06 - 0,52	5,8 ± 3	2,5	7,1	44,1	122,3
	Sagittal	Trunk	0,69	0,51 - 0,85	0,5 ± 0,3	0,2	0,4	30,4	84,4	0,53	0,31 - 0,74	0,5 ± 0,3	0,2	0,6	43,7	121,3
		Pelvis	0,68	0,49 - 0,84	1,6 ± 0,8	0,5	1,3	30,6	84,9	0,53	0,31 - 0,74	1,5 ± 0,9	0,6	1,8	42,6	118,1
		Hip	0,90	0,82 - 0,96	19 ± 8,1	2,6	7,3	13,8	38,2	0,83	0,7 - 0,92	18 ± 7	2,7	7,4	14,8	40,9
		Knee	0,71	0,53 - 0,86	35,5 ± 9,5	5,2	14,4	14,7	40,6	0,65	0,45 - 0,82	38,9 ± 9,2	5,4	14,9	13,9	38,4
		Ankle	0,65	0,45 - 0,82	17,5 ± 5,4	3,2	9,0	18,5	51,2	0,52	0,3 - 0,74	20,2 ± 6,2	4,3	11,9	21,3	59,0
between-session	Frontal	Trunk	0,79	0,47 - 0,92	0,8 ± 0,5	0,3	0,8	37,2	103,2	0,86	0,66 - 0,95	0,8 ± 0,4	0,2	0,6	27,6	76,6
		Pelvis	0,79	0,46 - 0,92	2,4 ± 1,6	0,9	2,5	36,3	100,7	0,86	0,64 - 0,94	2,3 ± 1,3	0,6	1,7	27,6	76,4
		Hip	0,76	0,41 - 0,91	7,1 ± 4,2	2,6	7,2	36,5	101,1	0,67	0,16 - 0,87	6,3 ± 3,2	2,3	6,3	36,4	101,0
		Knee	0,77	0,4 - 0,91	4,2 ± 2,2	1,4	3,8	33,3	92,4	0,69	0,19 - 0,88	4,4 ± 2,3	1,7	4,6	37,4	103,8
		Ankle	0,65	0,12 - 0,86	7 ± 3	2,0	5,4	27,8	77,1	0,68	0,22 - 0,87	7,3 ± 4	2,7	7,5	36,9	102,4
	Transverse	Trunk	0,78	0,45 - 0,91	0,5 ± 0,3	0,2	0,6	43,3	120,0	0,86	0,65 - 0,95	0,4 ± 0,2	0,1	0,3	32,0	88,6
		Pelvis	0,78	0,45 - 0,91	1,4 ± 1,1	0,6	1,8	44,4	123,0	0,85	0,61 - 0,94	1,2 ± 0,7	0,4	1,1	33,5	93,0
		Hip	0,78	0,43 - 0,91	6,2 ± 2,4	1,5	4,0	23,4	64,9	0,65	0,09 - 0,86	6,6 ± 2,2	1,7	4,6	25,0	69,4
		Knee	0,66	0,17 - 0,86	4,7 ± 1,9	1,4	3,8	28,9	80,2	0,36	0 - 0,75	4,7 ± 1,7	1,5	4,3	32,9	91,2
		Ankle	0,41	0 - 0,77	5,6 ± 2,2	1,9	5,4	34,8	96,6	0,60	0 - 0,84	5,9 ± 2,4	1,8	5,0	30,9	85,8
	Sagittal	Trunk	0,41	0 - 0,77	0,5 ± 0,3	0,3	0,7	49,9	138,4	0,46	0 - 0,79	0,5 ± 0,3	0,3	0,7	52,4	145,1
		Pelvis	0,39	0 - 0,76	1,6 ± 0,9	0,8	2,2	49,5	137,3	0,44	0 - 0,78	1,5 ± 0,9	0,8	2,1	52,0	144,0
		Hip	0,86	0,64 - 0,95	20,2 ± 8,5	3,9	10,7	19,1	53,0	0,86	0,63 - 0,94	19 ± 7,1	3,3	9,2	17,5	48,5
		Knee	0,79	0,47 - 0,92	35,7 ± 8,2	4,9	13,6	13,8	38,2	0,87	0,67 - 0,95	38,6 ± 7,8	3,8	10,6	9,9	27,4
		Ankle	0,81	0,52 - 0,93	17,4 ± 5,1	2,9	8,1	16,9	46,9	0,87	0,67 - 0,95	19,5 ± 4,8	2,3	6,3	11,6	32,1
between-operator	Frontal	Trunk	0,73	0,31 - 0,89	0,8 ± 0,6	0,3	1,0	41,7	115,7	0,75	0,39 - 0,9	0,8 ± 0,5	0,3	0,8	36,3	100,7
		Pelvis	0,73	0,31 - 0,89	2,5 ± 1,7	1,0	2,8	39,7	110,0	0,76	0,4 - 0,9	2,4 ± 1,4	0,9	2,4	35,5	98,4
		Hip	0,69	0,25 - 0,88	7,2 ± 4	2,7	7,5	37,4	103,6	0,54	0 - 0,82	6,5 ± 3,9	3,1	8,6	47,4	131,4
		Knee	0,78	0,47 - 0,91	4,6 ± 2,3	1,3	3,7	29,1	80,6	0,76	0,4 - 0,9	4,9 ± 2,4	1,5	4,2	30,9	85,5
		Ankle	0,79	0,49 - 0,92	6,4 ± 2,3	1,4	4,0	22,5	62,4	0,70	0,27 - 0,88	6,9 ± 3,2	2,1	5,9	30,9	85,8
	Transverse	Trunk	0,69	0,22 - 0,88	0,5 ± 0,3	0,2	0,6	44,0	121,9	0,52	0 - 0,81	0,4 ± 0,2	0,2	0,6	51,2	141,9
		Pelvis	0,71	0,28 - 0,89	1,4 ± 0,9	0,6	1,7	42,9	119,0	0,51	0 - 0,81	1,2 ± 0,7	0,6	1,7	50,0	138,5
		Hip	0,80	0,49 - 0,92	6 ± 2,1	1,3	3,7	21,9	60,8	0,77	0,41 - 0,91	6,7 ± 2,4	1,6	4,3	23,5	65,2
		Knee	0,83	0,56 - 0,93	4,5 ± 1,7	0,9	2,6	20,6	57,2	0,66	0,17 - 0,86	4,9 ± 2	1,4	3,9	29,1	80,6
		Ankle	0,84	0,6 - 0,94	5,2 ± 1,7	0,9	2,5	17,1	47,4	0,57	0 - 0,83	5,7 ± 2,1	1,7	4,6	29,1	80,6
	Sagittal	Trunk	0,70	0,22 - 0,88	0,5 ± 0,3	0,2	0,6	41,0	113,5	0,68	0,17 - 0,87	0,5 ± 0,2	0,2	0,5	35,2	97,5
		Pelvis	0,67	0,15 - 0,87	1,6 ± 0,9	0,6	1,7	40,5	112,3	0,68	0,18 - 0,78	1,5 ± 0,7	0,5	1,4	33,9	93,9
		Hip	0,86	0,65 - 0,94	19,8 ± 8,3	4,1	11,4	20,7	57,5	0,87	0,68 - 0,95	18,8 ± 7,5	3,5	9,6	18,5	51,2
		Knee	0,91	0,77 - 0,96	35 ± 8,5	3,5	9,7	10,0	27,7	0,91	0,79 - 0,97	38,5 ± 8,2	3,3	9,1	8,5	23,7
		Ankle	0,91	0,77 - 0,96	17,1 ± 4,9	2,0	5,6	11,8	32,7	0,85	0,64 - 0,94	19,6 ± 4,6	2,3	6,3	11,6	32,0

Table S2: Within-session, between-session and between-operator reliability (ICC and confidence intervals (CI)) and agreement (SEM, MDC, %SEM and %MDC) from the sit to stance (STS) task during both movement phases (Stand-to-sit and Sit-to-stand phase).

			Stand-to-sit							Sit-to-stand						
			ICC	CI	mean (± SD)	SEM	MDC	%SEM	%MDC	ICC	CI	mean (± SD)	SEM	MDC	%SEM	%MDC
within-session	Frontal	Trunk	0,84	0,72 - 0,93	0,8 ± 0,4	0,2	0,5	20,0	55,5	0,76	0,6 - 0,88	0,7 ± 0,4	0,2	0,5	24,6	68,3
		Pelvis	0,85	0,74 - 0,93	2,6 ± 1,3	0,5	1,4	18,7	52,0	0,63	0,43 - 0,81	2,3 ± 1	0,6	1,8	28,0	77,6
		Hip	0,92	0,85 - 0,96	6,4 ± 2,7	0,8	2,3	12,9	35,8	0,88	0,78 - 0,94	6,7 ± 2,7	1,0	2,6	14,2	39,4
		Knee	0,94	0,88 - 0,97	10,1 ± 3,7	1,0	2,7	9,5	26,4	0,96	0,92 - 0,98	10,9 ± 3,3	0,7	2,0	6,5	18,0
		Ankle	0,89	0,79 - 0,95	6,3 ± 2,9	1,0	2,7	15,4	42,8	0,83	0,7 - 0,92	6,6 ± 2,7	1,1	3,2	17,2	47,8
	Transverse	Trunk	0,82	0,69 - 0,92	0,7 ± 0,4	0,2	0,4	22,9	63,5	0,56	0,34 - 0,76	0,6 ± 0,3	0,2	0,6	33,3	92,3
		Pelvis	0,84	0,72 - 0,92	2,2 ± 1,2	0,5	1,3	20,5	56,9	0,56	0,35 - 0,76	2 ± 1	0,6	1,8	31,3	86,8
		Hip	0,93	0,87 - 0,97	8,9 ± 4,6	1,2	3,3	13,4	37,3	0,92	0,86 - 0,97	10,4 ± 4,5	1,3	3,6	12,5	34,8
		Knee	0,91	0,83 - 0,96	10,5 ± 3,9	1,2	3,3	11,3	31,5	0,97	0,94 - 0,99	9,7 ± 4,9	0,9	2,5	9,1	25,4
		Ankle	0,82	0,69 - 0,92	5,8 ± 2,2	1,0	2,8	17,6	48,7	0,86	0,75 - 0,93	6,1 ± 2,2	0,8	2,2	13,2	36,6
	Sagittal	Trunk	0,95	0,91 - 0,98	3,2 ± 1,9	0,4	1,1	12,3	34,0	0,96	0,93 - 0,98	3,7 ± 1,7	0,3	0,9	9,2	25,5
		Pelvis	0,96	0,92 - 0,98	9,7 ± 5,7	1,2	3,3	12,2	33,8	0,96	0,93 - 0,98	11,2 ± 5,3	1,0	2,9	9,2	25,6
		Hip	0,94	0,89 - 0,97	59 ± 8,3	2,6	7,3	4,5	12,3	0,93	0,88 - 0,97	60,5 ± 9,5	2,6	7,2	4,3	11,9
		Knee	0,96	0,93 - 0,98	57,7 ± 8	1,7	4,8	3,0	8,3	0,90	0,81 - 0,95	57,9 ± 9,1	2,9	7,9	4,9	13,7
		Ankle	0,93	0,87 - 0,97	11,2 ± 7,1	1,9	5,3	17,2	47,7	0,94	0,88 - 0,97	12,7 ± 7	1,7	4,7	13,5	37,4
between-session	Frontal	Trunk	0,56	0 - 0,82	0,8 ± 0,3	0,2	0,7	33,0	91,4	0,64	0,13 - 0,86	0,7 ± 0,3	0,2	0,6	32,7	90,5
		Pelvis	0,44	0 - 0,78	2,4 ± 1	0,9	2,4	34,9	96,8	0,57	0 - 0,83	2,2 ± 0,8	0,6	1,8	30,0	83,0
		Hip	0,00	0 - 0,51	6,2 ± 2,5	2,7	7,5	44,1	122,2	0,06	0 - 0,64	6,4 ± 2,5	2,4	6,6	37,2	103,0
		Knee	0,75	0,39 - 0,9	10,9 ± 4,7	2,8	7,8	25,9	71,7	0,64	0,11 - 0,85	11,4 ± 4,8	3,5	9,6	30,3	84,0
		Ankle	0,71	0,28 - 0,89	6,4 ± 2,5	1,7	4,8	26,9	74,6	0,78	0,45 - 0,91	6,8 ± 2,4	1,5	4,1	22,1	61,4
	Transverse	Trunk	0,26	0 - 0,56	0,7 ± 0,3	0,3	0,8	42,2	117,0	0,14	0 - 0,67	0,7 ± 0,3	0,3	0,7	40,1	111,3
		Pelvis	0,27	0 - 0,72	2,2 ± 1	0,9	2,4	39,3	109,0	0,31	0 - 0,73	2 ± 0,8	0,7	2,0	35,9	99,6
		Hip	0,72	0,33 - 0,89	8 ± 4	2,6	7,2	32,6	90,5	0,63	0,08 - 0,85	10 ± 4,9	3,6	10,0	35,8	99,4
		Knee	0,76	0,39 - 0,9	10,7 ± 5,2	3,3	9,1	30,7	85,0	0,85	0,61 - 0,94	9,5 ± 5,3	2,8	7,9	29,7	82,4
		Ankle	0,68	0,18 - 0,87	5,6 ± 2,4	1,8	5,0	32,1	88,9	0,75	0,38 - 0,9	5,5 ± 2,2	1,4	3,8	25,2	69,9
	Sagittal	Trunk	0,61	0,01 - 0,85	3 ± 1,5	1,2	3,2	38,5	106,6	0,63	0,06 - 0,85	3,4 ± 1,4	1,1	3,0	31,4	87,0
		Pelvis	0,60	0 - 0,84	9 ± 4,7	3,6	9,9	39,7	109,9	0,64	0,08 - 0,86	10,4 ± 4,4	3,3	9,0	31,1	86,2
		Hip	0,81	0,49 - 0,93	55,8 ± 8,9	5,1	14,2	9,2	25,4	0,86	0,63 - 0,95	58,2 ± 9,5	4,6	12,7	7,9	21,8
		Knee	0,94	0,85 - 0,98	57,9 ± 8,8	3,2	8,8	5,5	15,2	0,85	0,62 - 0,94	57,4 ± 8,1	4,4	12,3	7,7	21,4
		Ankle	0,69	0,22 - 0,88	11 ± 5,3	3,7	10,2	33,4	92,6	0,66	0,14 - 0,87	12,1 ± 5,3	3,7	10,2	30,4	84,2
between-operator	Frontal	Trunk	0,23	0 - 0,7	0,8 ± 0,3	0,3	0,9	39,5	109,4	0,34	0 - 0,75	0,7 ± 0,3	0,3	0,8	42,9	118,9
		Pelvis	0,24	0 - 0,71	2,5 ± 1	1,0	2,7	38,6	106,9	0,25	0 - 0,71	2,3 ± 0,9	0,9	2,5	39,3	108,9
		Hip	0,06	0 - 0,63	6,3 ± 2,9	5,9	16,4	94,3	261,4	0,14	0 - 0,66	6,3 ± 2,6	5,1	14,3	81,0	224,6
		Knee	0,77	0,41 - 0,91	10,3 ± 3,9	2,4	6,8	23,7	65,6	0,78	0,45 - 0,92	10,9 ± 3,5	2,2	6,0	20,0	55,3
		Ankle	0,76	0,38 - 0,91	6,1 ± 2,4	1,5	4,1	23,9	66,2	0,87	0,68 - 0,95	6,5 ± 2,3	1,1	3,1	17,4	48,2
	Transverse	Trunk	0,67	0,18 - 0,87	0,7 ± 0,4	0,3	0,8	40,4	112,1	0,30	0 - 0,72	0,7 ± 0,3	0,5	1,4	74,9	207,7
		Pelvis	0,51	0 - 0,8	2,3 ± 1,2	1,2	3,3	51,4	142,4	0,24	0 - 0,7	2,2 ± 1	2,0	5,5	92,1	255,3
		Hip	0,84	0,58 - 0,94	8,4 ± 4,1	2,2	6,2	26,6	73,7	0,51	0 - 0,8	10,4 ± 4	4,0	11,0	38,1	105,6
		Knee	0,84	0,59 - 0,94	10,2 ± 4,6	2,5	6,8	24,0	66,4	0,87	0,66 - 0,95	9,4 ± 5,1	2,5	6,8	26,3	72,8
		Ankle	0,76	0,41 - 0,9	5,6 ± 1,9	1,3	3,5	22,6	62,6	0,72	0,31 - 0,89	5,7 ± 2	1,3	3,6	22,8	63,1
	Sagittal	Trunk	0,24	0 - 0,71	3,1 ± 1,6	1,4	3,9	46,2	128,1	0,38	0 - 0,76	3,5 ± 1,6	1,4	3,7	38,1	105,7
		Pelvis	0,27	0 - 0,72	9,3 ± 4,9	4,3	12,0	46,4	128,7	0,41	0 - 0,77	10,8 ± 4,7	4,1	11,5	38,3	106,2
		Hip	0,73	0,34 - 0,89	57,2 ± 9,6	7,9	21,9	13,8	38,3	0,77	0,45 - 0,91	58,7 ± 10,3	7,9	22,0	13,5	37,5
		Knee	0,93	0,83 - 0,97	57,6 ± 7,7	3,4	9,4	5,9	16,3	0,86	0,64 - 0,95	57,6 ± 8,2	4,5	12,4	7,8	21,6
		Ankle	0,89	0,72 - 0,96	10,6 ± 5,8	2,6	7,1	24,4	67,7	0,86	0,65 - 0,94	11,9 ± 6,2	2,9	8,0	24,2	67,1

Figure S1: Waveforms of the single leg squat (SLS) task of all participants (n=20) from the trunk, pelvis, hip, knee and ankle, abd-adduction, int-external rotation and flexion-extension angles during the flexion phase and extension phases, from the three sessions: day1-operator1 (solid); day1-operator2 (striped); day2-operator1 (dotted).

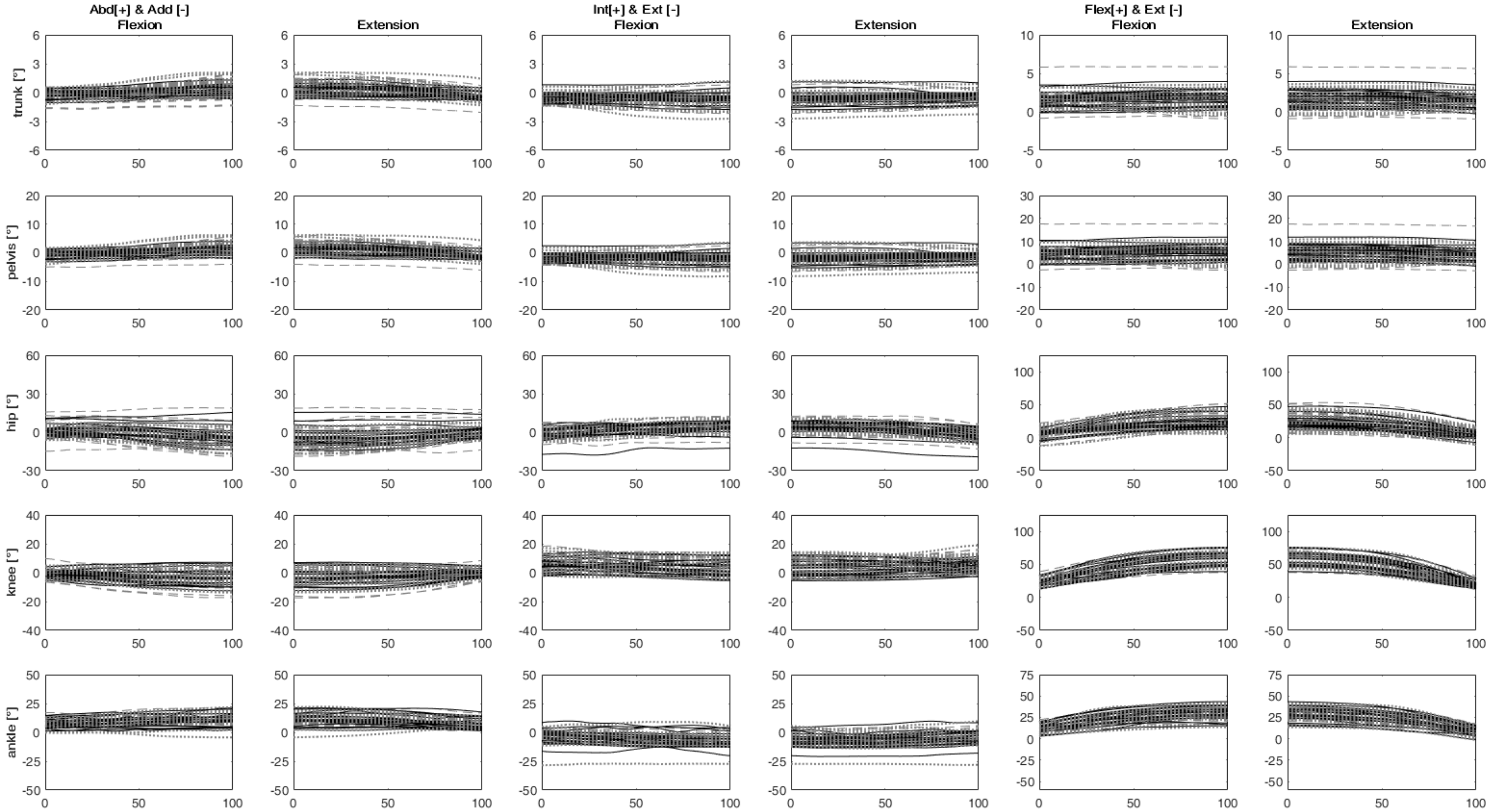

Figure S2: Waveforms of the sit to stand task (STS) of all participants (n=20) from the trunk, pelvis, hip, knee and ankle, abd-adduction, int-external rotation and flexion-extension angles during the flexion phase and extension phases, from the three sessions: day1-operator1 (solid); day1-operator2 (striped); day2-operator1 (dotted).

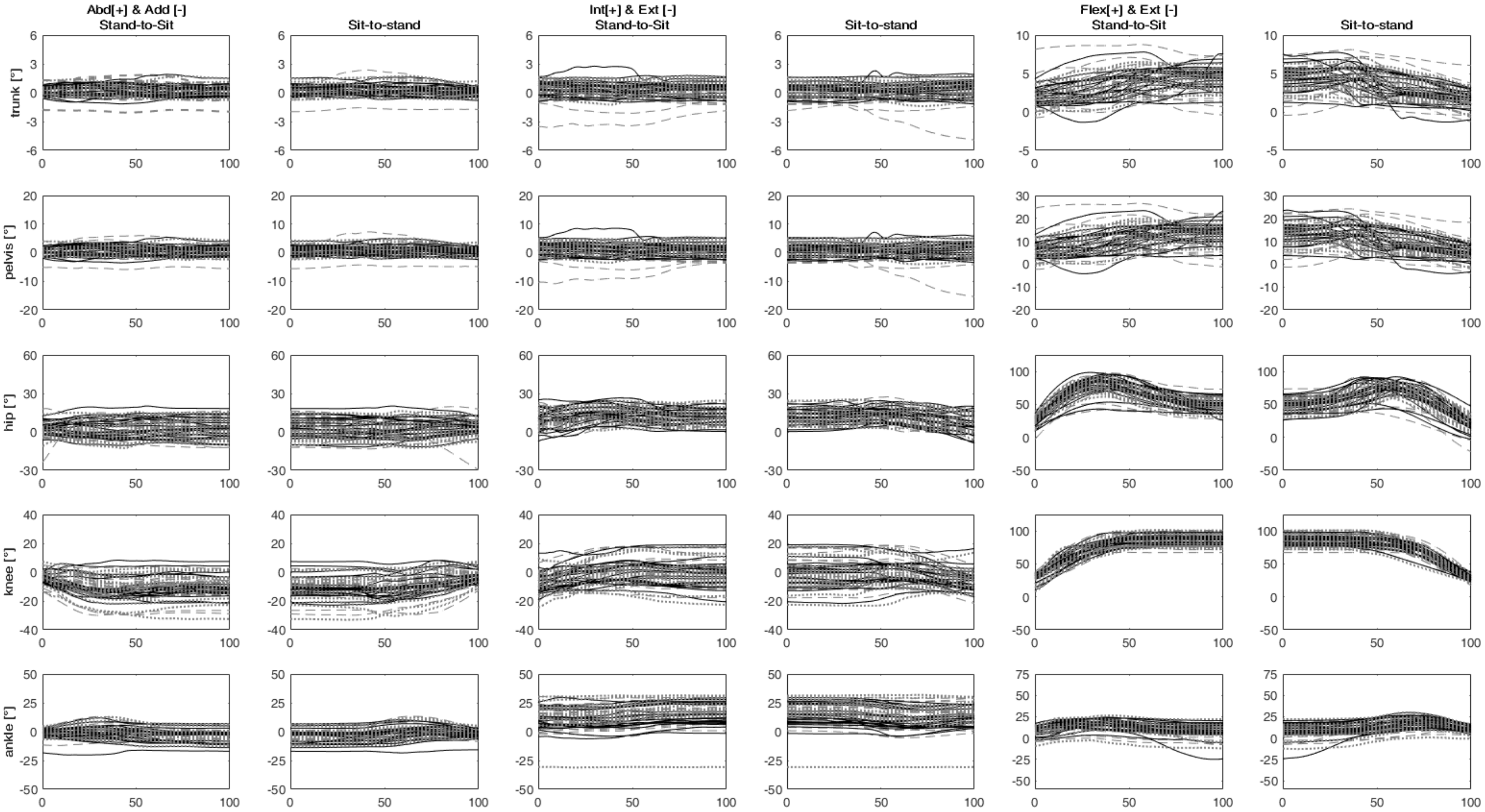

Supplement: Supplementary file 1 [file sensors-19-00141-s001.pdf]
